# The Associations of Exposome Score with Various Domains of Psychopathology: A Network Analysis in a Non-Clinical Sample

**DOI:** 10.3390/brainsci14030242

**Published:** 2024-02-29

**Authors:** Maksymilian Rejek, Błażej Misiak

**Affiliations:** Department of Psychiatry, Wroclaw Medical University, 50-367 Wroclaw, Poland; maksymilian.rejek@gmail.com

**Keywords:** psychosis, mood disorder, depression, transdiagnostic psychiatry, early intervention

## Abstract

Background: The intricate correlation between environmental exposures and mental health outcomes is increasingly acknowledged in psychiatric research. This study investigated the relationship between cumulative environmental risk factors, as represented by the exposome score (ES), and various domains of psychopathology within a non-clinical sample using a network analysis. Methods: We recruited 1100 participants (aged 18–35 years, 51.4% females) via a computer-assisted web interview, assessing psychopathological symptoms using standardized questionnaires. Environmental exposures, including season of birth, obstetric complications, advanced paternal age, childhood trauma, cannabis use, and urban upbringing, were self-reported to calculate the ES. Results: A network analysis revealed significant associations of the ES with psychotic-like experiences (PLEs) (weight = 0.113), manic (weight = 0.072), and attention-deficit/hyperactivity disorder symptoms (weight = 0.062). These connections did not differ significantly with respect to their weights. Depressive symptoms had the highest centrality and predictability. The mean predictability across all nodes included in the network was 0.344. Conclusions: These findings underscore the transdiagnostic nature of environmental exposures, aligning with previous research indicating broad associations between the ES and various facets of psychopathology. Our results suggest that the ES may not specifically correlate with PLEs but may indicate the risk of a broader psychopathology.

## 1. Introduction

In the ever-evolving field of psychiatric research, there is a growing emphasis on unraveling the complex interplay between environmental exposures and mental health outcomes. Over the course of a lifetime, individuals encounter a myriad of environmental factors. These exposures, such as season of birth, obstetric complications, advanced paternal age at conception, growing up in an urban environment, and the experience of childhood trauma have the potential to heighten the risk of developing psychopathology, particularly in those with pre-existing vulnerabilities [1,2]. Moreover, the influence of chronic stress, especially during critical neurodevelopmental stages, is emerging as a powerful catalyst in shaping mental health outcomes [3,4]. There is a growing and active interest in unraveling the intricate relationship between mental disorders and environmental exposures, given their modifiable and potentially preventable nature.

Traditionally, psychiatric research has focused on examining the effects of individual environmental exposures on specific mental health outcomes [5]. Nonetheless, a paradigm shift is underway, acknowledging that these factors are not limited to specific phenotypes, with certain risk factors contributing to a spectrum of mental disorders. In addition to genetic susceptibility, various disorders may stem from different risk factors. Many environmental factors can affect various behavioral phenotypes. For example, cannabis abuse has been linked to elevated risks of psychosis spectrum disorders as well as anxiety, depression, and bipolar disorders [6]. Also, studies have indicated that individuals who experienced childhood trauma are more likely to develop non-specific psychopathology manifesting in affective, anxiety, and psychotic symptoms that transcend traditional diagnostic categories. This aligns with findings from a recent umbrella review, which demonstrated that a history of childhood trauma is linked with various mental disorders [7]. Likewise, urbanicity, advanced paternal age, and obstetric complications have been identified to show transdiagnostic associations [8,9,10,11,12]. Environmental exposures are intricately entwined, showcasing both causative and non-causative associations, along with complex interactions. For instance, cannabis use, recognized as an environmental risk factor for psychosis, often co-occurs with other exposures including childhood adversity and urbanicity. Previous research has suggested that exposure to environmental risk factors increases connectivity across the symptom network [6]. These exposures show correlations and mutual interactions, while research also indicates a dose-response correlation between exposures and psychopathological outcomes, wherein a greater number or severity of exposures tends to correlate with progressively worse outcomes [13,14].

A novel exploration that aims to better understand the association between environmental risk factors and psychopathology is related to investigating cumulative measures that comprehensively encompass multiple risk factors. These measures are known as the exposome score (ES) or polyenviromic risk score (PERS) [15,16]. The ES, akin to risk scores in other medical disciplines, aims to include various environmental factors associated with mental disorders. Researchers have used various methods to explore the collective impact of various environmental exposures linked to mental disorders [13,17,18,19]. The predominant approach involves a straightforward total score of environmental risk loading, often derived by summing individual factors. However, this method fails to recognize the varying risk magnitudes associated with each exposure [20]. To address this limitation, prior research has turned to estimates from meta-analyses to formulate a weighted environmental sum score, which is a method that exerts conceptual parallels with the polygenic risk score [21]. A recent advancement in this field is the ES for schizophrenia, a cumulative environmental exposure score tailored to psychotic disorders. Previous studies have demonstrated the potential utility of these scores in predicting the risk of developing psychosis, with the ES explaining a greater amount of variance in psychosis risk than genetic factors alone [22]. It is crucial to note the complexity of constructing these scores, as each score represents a unique combination of environmental factors. The particular exposures incorporated into each score, along with their relative weights, may differ based on the population and circumstances. Beyond its associations with the extended psychosis phenotype, a higher ES for schizophrenia has shown links with poorer mental and physical outcomes.

To date, it remains unknown as to whether the ES is specifically associated with PLEs in non-help-seeking individuals. Some studies employing the ES suggest that the vulnerability, assessed through the exposome, is associated not only with psychotic spectrum disorders but also with a wider range of mental health problems and functional impairment [23]. This observation is consistent with the understanding that psychosis manifests along a continuum and overlaps with other symptom dimensions [17,24]. Additionally, reports indicating that psychosis expression may be preceded by a nonspecific prodrome with mixed psychopathology domains could also be relevant [25,26]. In our previous studies, initially, we focused on investigating the association between psychotic-like experiences and a broad spectrum of psychopathology. Subsequently, our aim was to explore whether the exposome score (ES) may also be linked to the extended psychosis phenotype and its individual symptoms. Specifically, we identified that the ES is associated with positive screening for psychosis using the Prodromal Questionnaire–16 (PQ–16) in a non-clinical sample [27]. With respect to single PQ–16 items, we found that the ES is associated not only with PLEs (i.e., “paranoid thoughts”, “a lack of control over own ideas or thoughts”, “thought echo”, and “being distracted by distant sounds”) but also items covering depressive and anxiety symptoms (“uninterested in things used to enjoy” and “feeling anxious when meeting people for the first time”). In the present study, we aimed to further explore which domains of psychopathology are associated with the ES score in a community sample of non-help-seeking individuals.

## 2. Materials and Methods

### 2.1. Participants

Participants were recruited through the computer-assisted web interview conducted in March 2023 and available on the online survey platform dedicated to research purposes. Only registered users had access to the survey. The survey was based on two specific inclusion criteria: age between 18 and 35 years and a self-reported absence of prior psychiatric treatment. Recruitment procedures adhered to the sociodemographic composition of the Polish population in 2021, encompassing 51% males, 34% of participants aged 18–24 years, and 60% residing in urban areas (i.e., 32% living in cities with populations between 100,000 and 200,000 inhabitants, 9% living in cities with populations between 200,000 and 500,000: 7%, and cities with populations exceeding 500,000: 12%). Prior to responding to the survey, participants were apprised of its confidential and anonymous nature. Certain results obtained from this dataset were published previously [27,28]. This research received approval from the Bioethics Committee at Wroclaw Medical University, Wroclaw, Poland (approval number: 99/2023).

### 2.2. Assessment of Psychopathology 

A detailed description of questionnaires used to assess psychopathological symptoms with corresponding measures of internal consistency is provided in Supplementary Table S1. In brief, we used the following questionnaires: (1) the Patient Health Questionnaire–9 (PHQ–9) for depressive symptoms [29]; (2) the Mood Disorder Questionnaire (MDQ) for manic symptoms [30,31]; (3) the Obsessional Compulsive Inventory-Revised (OCI-R) for obsessive-compulsive disorder (OCD) symptoms [32]; (4) the Generalized Anxiety Disorder–7 (GAD–7) for anxiety symptoms [33]; (5) the PQ–16 for PLEs [34] and (6) the Adult ADHD Self-Report Scale for DSM-5 (ASRS-5) for ADHD symptoms [35].

### 2.3. Assessment of the ES

The following exposures were assessed using self-reports: (1) winter season of birth; (2) obstetric complications; (3) advanced paternal age; (4) handedness; (5) a history of childhood trauma (emotional neglect, emotional abuse, bullying, and sexual abuse); (6) problematic cannabis use; and (7) urban upbringing (Table 1). To calculate the ES, we adhered to the methods proposed previously [16]. Specifically, all exposures were binarized and multiplied by the log odds derived from previous meta-analyses. Next, they were summed and divided by the number of exposures recorded in this study. The log odds were almost the same as those used in the study by Padmanabhan, Shah, Tandon, and Keshavan [16]. For the winter season of birth, the log odds from an updated meta-analysis were used [36]. Handedness was not included in the study by [16]. Therefore, the log odds from the meta-analysis by Hirnstein and Hugdahl [37] were used. 

### 2.4. Statistics

Data were analyzed by using network analysis techniques implemented within the R software (version 4.1.3). Variables of interest had approximately normal distribution as absolute values of skewness and kurtosis were lower than 2 and 4, respectively. Variables included in the network covered symptom scores and covariates (age, gender, education, and employment status). The network was estimated using the Mixed Graphical Models (the *mgm* package) as we included continuous (symptom scores and age) and binary variables (education—higher vs. other than higher, gender, employment status—employed vs. unemployed) in the network [38]. To improve the interpretability of the network, the L1-penalized regression (LASSO) was used [39,40]. The LASSO shrinks partial correlations with low coefficients. The node centrality was estimated by calculating the node strength. The node strength is the sum of all edge weights connected to it. Also, the node predictabilities were estimated. The predictability provides information about the proportion of variance explained by nodes directly connected to it. Visualizations were carried out using the *qgraph* package [41]. 

The final stage of a network analysis was performed in the *bootnet* package [42]. It was used to evaluate bootstrapped differences in edge weights and node centralities together with network accuracy and stability. Case-drop bootstrapping, consisting of 1000 iterations, was performed to assess the stability of node strength. Additionally, non-parametric bootstrapping involving 1000 iterations was employed to evaluate the stability of edge weights. The network stability was explored by computing the correlation stability coefficient (CS-C), which should exceed 0.25.

**Table 1:** Components of the exposome score.

Exposure	Assessment	**Odds Ratio [Log Odds Ratio]**
Winter season of birth	Respondents were requested to report their month of birth, and then, based on the meteorological seasons in the Northern Hemisphere, individuals born between December and February were assigned to the winter season, as reported in the recent meta-analysis by Coury, Lombroso, Avila-Quintero, Taylor, Flores, Szejko, and Bloch [36].	1.05 [0.05] [36]
Obstetric complications	The history of obstetric complications was documented using the following questions: “Were you delivered by caesarean section due to perinatal complications?”, “Was your birth weight less than 2500 g?”, and “Were you delivered preterm, i.e., before the 37th week of pregnancy?”. Possible responses were “yes”, “no”, and “I don’t know”. Respondents were categorized as having exposure to obstetric complications if their response to any of these questions was “yes”. Responses of “I don’t know” were coded as missing data.	2.00 [0.69] [11]
Advanced paternal age	Participants were asked to report the age of their father at the time of their birth. Advanced paternal age was defined as ≥35 years.	1.28 [0.25] for paternal age of 35–54 years and 2.22 [0.80] for ≥55 years [43]
Handedness	Participants were queried about their hand preference, i.e., whether they are right-handed, left-handed, or have mixed-handedness. Subsequently, in line with a prior meta-analysis [43], responses indicating left-handedness and mixed-handedness were collectively analyzed as non-right-handedness.	1.65 [0.50] [37]
Childhood trauma	Participants were screened for a history of emotional neglect, emotional abuse, bullying, and sexual abuse before the age of 17. Three questions from the Traumatic Experience Checklist (TEC) [44] were used to assess emotional neglect, abuse, and bullying: “When you were a child or a teenager, have you ever felt emotionally neglected (e.g., being left alone, insufficient affection) by your parents, brothers or sisters?”; “When you were a child or a teenager have you ever felt emotionally abused (e.g., being belittled, teased, called names, threatened verbally, or unjustly punished) by your parents, brothers or sisters?”, and “When you were a child or teenager, did you experience psychological violence (e.g., nicknames, teasing) or physical abuse (e.g., jerking, beating) from your peers?”. A history of sexual abuse was assessed using the following questions from the Childhood Experience of Care and Abuse (CECA.Q) [45,46]: “When you were a child or teenager did you have any unwanted sexual experiences?”; “Did anyone force you or persuade you to have sexual intercourse against your wishes before age 17?” and “Can you think of any upsetting sexual experiences before age 17 with a related adult or someone in authority e.g., teacher?”. Participants who confirmed a history of any of these experiences were classified as having been exposed to sexual abuse.	Emotional neglect: 2.90 [1.06], emotional abuse: 3.40 [1.22], bullying: 2.39 [0.87], and sexual abuse: 2.38 [0.87] [47]
Problematic cannabis use	To identify problematic cannabis use, we applied 11 of the 16 questions of the Cannabis Problems Questionnaire (CPQ) [48], which refer to the past 12 months: “Have you tended to smoke more on your own than you used to?”; “Have you been neglecting yourself physically?”; “Have you felt depressed for more than a week?”; “Have you been so depressed you felt like doing away with yourself?”; “Have you given up recreational activities you once enjoyed for smoking?”; “Do you find it hard to get the same enjoyment from your usual interests?”; “Have you felt more antisocial after smoking?”; “Have you worried about getting out of touch with friends or family?”; “Have you been concerned about a lack of motivation?”; “Have you worried about feelings of personal isolation or detachment?” and “Do you usually have a smoke in the morning, to get yourself going?”. Respondents who reported at least one of these consequences of cannabis use were identified as showing problematic cannabis use.	1.75 [0.56] [49]
Urban upbringing	Participants were asked to categorize their primary residence according to the following classifications: (1) rural; (2) a city of up to 100,000 inhabitants; (3) a city of 200,000–500,000 inhabitants; and (4) a city over 500,000 inhabitants. In the data analysis process, the responses were categorized into those reporting a rural or urban place of residence.	1.72 [0.54] [8]

## 3. Results

The sample characteristics are summarized in Table 2. There were 1100 participants (aged 27.1 ± 5.1 years, 48.6% males). Most frequently, they reported secondary education (50.3%) and full-time employment (51.3%).

**Figure 1:**
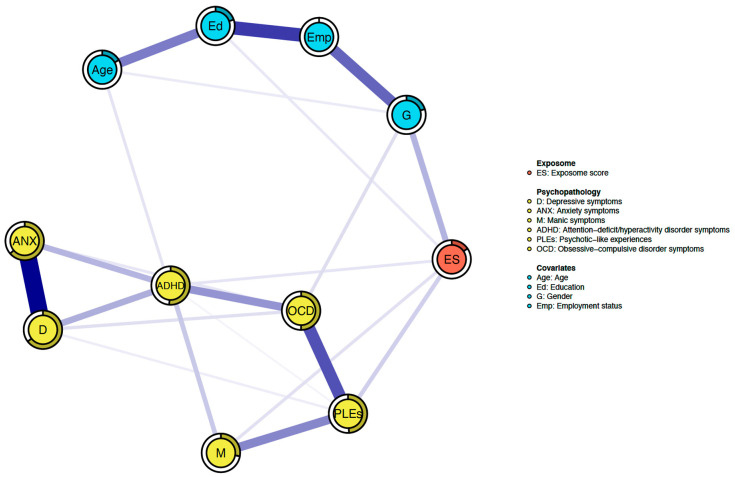
The network assessed in the present study. Specific variables are shown as nodes that are connected by edges. All edges show positive associations. Thicker edges refer to stronger associations. The filled parts of rings around nodes illustrated node predictability.

All nodes in the network were well-connected and no negative edges were found (Figure 1, Table 3). A total of 22 edges (out of 55 potential edges) had non-zero weights (40.0%). The ES node was directly connected to three nodes representing symptom scores, including PLEs (weight = 0.113), manic symptoms (weight = 0.072), and ADHD symptoms (weight = 0.062). These edges did not differ significantly with respect to their weights (Supplementary Figure S1). The highest strength centrality was obtained for the node representing depressive symptoms, while the lowest one was found for the ES (Supplementary Figure S2). The strength centrality of depressive symptoms was significantly higher compared to the strength centrality of the ES and manic symptoms, but not in comparison with other nodes (Supplementary Figure S3). The mean predictability of the whole network was 0.344. This means that on average the network explained 34.4% of the variance in the included variables (nodes). Apart from covariates, depressive symptoms had the highest predictability (0.646), while the ES had the lowest predictability (0.155) (Supplementary Table S2). 

Bootstrapping procedures demonstrated that the network analysis had sufficient stability and accuracy (Supplementary Figures S4 and S5). The CS-C values for the strength centralities and edges were 0.672 and 0.750, respectively. 

## 4. Discussion

In this study, we sought to unravel the intricate correlation between the ES and diverse domains of psychopathology within a non-clinical sample. Building upon our prior findings [27], which linked the ES not only to PLEs but also to experiences representing anxiety and depressive symptoms, indicating the contributory role of ES in non-specific psychopathology. The present analysis provides a deeper insight into specific domains of psychopathology. Our findings reveal significant associations between the ES and specific psychopathological domains, including PLEs, manic, and ADHD symptoms. These advancements contribute to a more nuanced comprehension of the ES’s influence on shaping mental health outcomes. The results align with the evolving paradigm in psychiatric research, underscoring the transdiagnostic nature of environmental exposures. 

Previous research indicates that the ES may not only be associated with an elevated risk of psychosis expression within the extended psychosis phenotype but also correlates with the severity of functional impairment [43]. Other studies have revealed a link between the ES and a broad spectrum of mental and physical health outcomes, encompassing conditions such as depression, anxiety, alcohol use disorder, as well as various somatic health impairments [44]. The study conducted by Pries et al. showed the strongest association of the ES with schizophrenia, followed by bipolar disorder and suicidal ideation [45]. These findings propose that environmental exposures are not exclusively linked to the psychosis spectrum phenotype but, instead, show a more widespread association with various facets of psychopathology. In our study, the ES node was directly connected to three nodes representing PLEs, manic, and ADHD symptoms. Such results imply the transdiagnostic and multidimensional nature of the ES. Despite differences in the nature of these symptoms, the edges connecting the ES node to symptom nodes did not differ significantly in terms of their weights. This suggests a relatively uniform influence of the ES on various domains of psychopathology. These results align with and expand upon the recently proposed conceptualization of psychopathology as a network comprising causally linked sets of symptoms in which symptoms reciprocally influence each other over time, culminating in the development of a more defined syndrome characterized by a specific pattern within a dynamic network for each syndrome [6]. The prior study utilized a network analysis of general population data to elucidate the association between environmental risk factors (specifically, cannabis use, trauma, and urban environment) and various dimensional measures of psychopathology (including anxiety, depression, OCD symptoms, and a composite measure of psychosis expression) [46]. The results unveiled specific pathways connecting environmental factors to symptoms, with cannabis use often playing a significant role in these pathways. Additionally, the analyses suggest that individuals exposed to environmental risk factors demonstrate stronger connections within symptom networks, implying that environmental exposures might play a role in fostering less resilient symptom networks. Moreover, environmental factors have been shown to heighten the probability of psychosis expression by increasing general psychopathology [47]. Environmental exposure leads to a more strongly connected and consequently more susceptible psychopathology network. Several studies within the general population have revealed that symptom domains are interconnected and do not occur independently, even prior to the onset of a specific disorder. Furthermore, these studies indicate that interactions between symptoms can forecast the progression to a more severe mental health outcome [48,49,50]. It has also been shown that exposure to environmental risk factors (including trauma, urban living, and cannabis use) enhances the connectivity between symptoms associated with affective dysregulation and the expression of psychosis in a dose-response manner [23]. This aligns with the theory that disturbances induced by the environment spread through the network of psychopathology, leading to an increased mixture of psychosis symptoms and a gradual expansion and strengthening of connectivity. This progression might ultimately result in the transition to a more severe, distinct clinical syndrome.

Centrality metrics play a crucial role in comprehending network analysis, offering insights into the importance of individual nodes within the network. The network approach facilitates the identification of central nodes, which are highly interconnected. In our study, the node representing depressive symptoms demonstrated the highest strength centrality and predictability, indicating its substantial influence and strong connections with other nodes. This aligns with findings from other studies where depressive symptoms were noted either as the most central or bridging symptoms within broader psychopathology [51,52,53]. Additionally, depressive symptoms exhibit robust associations, extending beyond mental health to include physical health outcomes and a general propensity for medical comorbidity [54]. Previous research suggests that central nodes are pivotal intervention targets due to their ability to have a more profound impact on altering other nodes [55]. Centrality measures arrange nodes based on their connections, while predictability, although similar in concept, offers absolute values indicating the percentage of variance explained by other variables in the network [56]. Nodes with high predictability and centrality are deemed key variables for potential interventions, suggesting that targeting depressive symptoms may have the most significant impact on associated psychopathological domains. Conversely, the ES node exhibited the lowest strength in centrality and predictability, implying a comparatively weaker direct influence on other nodes.

While our study provides valuable insights into the association between the ES and psychopathology, it is essential to acknowledge certain limitations. First, the cross-sectional nature of our data restricts our ability to establish causality. The observed relationships between the ES and specific psychopathological domains do not imply a directional influence, and longitudinal studies are warranted to elucidate the temporal dynamics of these associations. Also, the accuracy of data obtained using internet-based surveys might be limited. Moreover, the specific characteristics of our non-clinical sample may constrain the applicability of our results to other populations, including those with more severe psychopathology. Subsequent studies should delve into the enduring impacts of the ES on psychopathology over time and encompass a more extensive array of environmental exposures. Furthermore, depending on self-reported evaluations for both environmental risk factors and psychopathological symptoms introduces the risk of recall bias and subjective interpretation, particularly regarding obstetric complications. Nevertheless, in case of obstetric complications, our survey allowed participants to express uncertainty regarding their exposure history, and we subsequently excluded such cases from the data analysis. It is also noteworthy to highlight that standardized tools were not employed for assessing handedness. Regarding the CPQ, only specific items were utilized. Similarly, when documenting a history of childhood trauma, we utilized specific items from the CECA.Q and TEC. Moreover, the use of dichotomized risk factors within the ES might have led to the loss of information. It is worth mentioning that our study was conducted among a non-help-seeking population, mainly consisting of young adults, where some individuals, even with exposomic vulnerability, may not develop distinct mental health problems later in life. Also, the use of a non-clinical sample without assessment of psychopathology using validated diagnostic instruments may limit the generalizability of our results to clinical populations. Furthermore, various limitations related to the snowball method and sampling accuracy should be taken into consideration. It is essential to consider the potential overlap of psychopathological symptoms. For example, the symptoms assessed by the ASRS-5 could potentially reflect cognitive impairments associated with PLEs, mood, or OCD symptoms. Similarly, symptoms from the MDQ might overlap with those related to borderline personality disorder. Moreover, the assessment of environmental exposures and psychopathological symptoms is inherently complex, and the inclusion of additional relevant factors (such as ethnic minority, migration, and pregnancy complications) or refining exposure assessments may enhance the precision of future studies. In conclusion, while our study contributes valuable insights, future research should address these limitations to foster a more comprehensive understanding of the complex interplay between the exposome, psychopathology, and mental health outcomes.

In summary, our findings indicate that the ES may not be specifically associated with the occurrence of PLEs in non-help-seeking individuals. This observation might be informative for future studies on the ES, suggesting that risk stratification, based on cumulative measures, should rather focus on investigating broader mental health outcomes. This is of particular importance as the majority of environmental exposures show non-specific associations with distinct aspects of psychopathology. These findings also hold potential implications from the public health perspective suggesting that comprehensive interventions focused on early-life vulnerabilities might improve outcomes of individuals at risk of developing psychopathology. Reducing the ES might be achieved by targeting modifiable risk factors, e.g., problematic cannabis use. In case of other exposures, e.g., a history of childhood trauma, therapeutic interventions might reduce their long-lasting effects. 

## Figures and Tables

**Table 2:** Sociodemographic and clinical characteristics of the sample.

	Mean ± SD or *n* (%)
Age, years	27.1 ± 5.1
Gender, males	535 (48.6)
Education	
Primary	61 (5.5)
Vocational	89 (8.1)
Secondary	553 (50.3)
Higher	397 (36.1)
Occupation	
Unemployed	164 (14.9)
Part–time	170 (15.5)
Student	202 (18.4)
Full–time	564 (51.3)
Urbanicity, urban	672 (61.1)
Winter season of birth	269 (24.5)
Advanced paternal age	223 (24.8) *
Obstetric complications	276 (31.9) **
Handedness, non-right	107 (9.7)
Emotional neglect	576 (52.4)
Emotional abuse	477 (43.4)
Bullying	593 (53.9)
Sexual abuse	233 (21.2)
CPQ, any cannabis-related problems	72 (6.5)
PQ–16	5.3 ± 4.0
GAD–7	7.6 ± 5.5
PHQ–9	9.4 ± 6.2
MDQ	5.5 ± 3.7
OCI–R	22.3 ± 14.7
ASRS-5	10.1 ± 4.3
ES	0.25 ± 0.17

Note: ASRS-5; the Adult ADHD Self–Report Scale for DSM-5; ES, the exposome score; GAD–7, the General Anxiety Disorder–7; MDQ, the Mood Disorder Questionnaire; OCI–R, the Obsessive-Compulsive Inventory–Revised; PHQ–9, the Patient Health Questionnaire–9; PQ–16, the Prodromal Questionnaire–16. * *n* = 200 (18.2%) with missing data ** *n* = 236 (21.5%) with missing data.

**Table 3:** Edge weights.

	ES	D	ANX	M	ADHD	PLEs	OCD	Age	Ed	G
D	0.000	-								
ANX	0.000	0.590	-							
M	0.072	0.000	0.000	-						
ADHD	0.062	0.191	0.175	0.128	-					
PLEs	0.113	0.046	0.000	0.279	0.027	-				
OCD	0.000	0.077	0.061	0.000	0.246	0.398	-			
Age	0.000	0.000	0.000	0.065	0.000	0.000	0.000	-		
Ed	0.057	0.000	0.000	0.000	0.000	0.000	0.000	0.304	-	
G	0.177	0.000	0.000	0.000	0.000	0.000	0.082	0.052	0.000	-
Emp	0.000	0.000	0.000	0.000	0.000	0.000	0.000	0.000	0.445	0.355

Note: ADHD, attention-deficit/hyperactivity disorder symptoms; ANX, anxiety symptoms; D, depressive symptoms; Ed, the level of education; Emp, employment status; ES, the exposome score; G, gender; M, manic symptoms; OCD, obsessive-compulsive disorder symptoms; PLEs, psychotic-like experiences.

## Data Availability

Data that are the basis of the present study are available from the corresponding author upon reasonable request. The data are not publicly available due to privacy and ethical restrictions.

## Supplementary Materials

The following supporting information can be downloaded at: https://www.mdpi.com/article/10.3390/brainsci14030242/s1, Table S1: Questionnaires used to assess psychopathological symptoms; Table S2: Node predictabilities; Figure S1: Bootstrapped differences between edge weights in the network analyzing symptoms associated with the exposome score; Figure S2: Strength centralities; Figure S3: Bootstrapped differences in the strength centrality; Figure S4: Stability of the strength centrality index; Figure S5: Bootstrapped 95% confidence intervals of edge weights.
