# Supplementary material for: The Associations of Exposome Score with Various Domains of Psychopathology: A Network Analysis in a Non-Clinical Sample
Source: Brain Sci. 2024 Feb 29;14(3):242. doi: 10.3390/brainsci14030242 (PMC10968955; doi:10.3390/brainsci14030242)
Supplement: Supplementary file 1 [file brainsci-14-00242-s001.zip › brainsci-2888722-supplementary.pdf]

Supplementary Material

Table S1. Questionnaires used to assess psychopathological symptoms.

**Symptoms**	**Questionnaire**	**Cronbach's alpha**
Depressive symptoms	We evaluated depressive symptoms using the Patient Health Questionnaire-9 (PHQ-9) [1]. This questionnaire assesses the presence of depressive symptoms in the preceding 2 weeks. Items are rated on a 4-point scale (responses ranging from 0 - 'not at all' to 3 - 'nearly every day'). The PHQ-9 score ranges between 0 and 27. Higher scores are equivalent to higher levels of depressive symptoms.	0.878
Manic symptoms	To assess the presence of manic symptoms, we employed the Mood Disorder Questionnaire (MDQ) [2,3]. It consists of 13 items that measure the presence of manic symptoms over a lifetime, using a binary scale for responses (yes or no). Two additional questions inquire about the simultaneous presence of at least two symptoms and associated impairment. In the present study, we focused on the level of lifetime manic symptoms. Therefore, the total MDQ score in our study ranged between 0 and 13.	0.840
Anxiety symptoms	Assessment of anxiety symptoms was conducted using the Generalized Anxiety Disorder-7 (GAD-7) questionnaire [4]. It consists of seven items that measure the presence of anxiety symptoms over the preceding 2 weeks, with responses recorded on a 4-point scale (ranging from 0 - "not at all," to 3 - " nearly every day"). The total GAD-7 score ranges from 0 to 21. Higher scores are equivalent to higher levels of anxiety symptoms.	0.925
OCD symptoms	To assess the occurrence of symptoms related to OCD, we employed the Obsessional Compulsive Inventory-Revised (OCI-R). The OCI-R consists of 18 items that examine the level of distress associated with symptoms of obsessive-compulsive disorder and hoarding disorder over the preceding month. All responses are measured on a 5-point scale ranging from 0 ("not at all") to 4 ("extremely"). The total score of the OCI-R ranges from 0 to 72. Higher scores are equivalent to higher levels of OCD symptoms.	0.929
PLEs	To assess PLEs, we used the Prodromal Questionnaire-16 (PQ-16), which was originally designed to identify individuals at risk of psychosis [5]. It contains 14 items measuring the presence of psychotic experiences (true-or-false responses) and 2 items that are most closely related to anxiety and depressive symptoms (items 1 and 7). This study assessed PLEs over the preceding 4 weeks. Due to the overlap of symptoms captured by items 1 and 7 with depressive and anxiety symptoms, these items were excluded from calculating the total score. Therefore, the total score ranged between 0 and 14. Higher scores are equivalent to higher levels of PLEs.	0.843
ADHD	Symptoms of ADHD were assessed using the Adult ADHD Self-Report Scale for DSM-5 (ASRS-5) [6]. This is a 6-item questionnaire that measures symptoms over the preceding 6 months. Items are scored on a 5-point scale ranging from 0 ("never") to 4 ("very often"). The total score ranges from 0 to 24. Higher scores are equivalent to higher levels of ADHD symptoms.	0.756

Note: ADHD, attention-deficit/hyperactivity disorder; OCD, obsessive-compulsive disorder; PLEs, psychotic-like experiences

Table S2. Node predictabilities.

Node name	Predictability
ES	0.155
D	0.646
ANX	0.637
M	0.278
ADHD	0.512
PLEs	0.492
OCD	0.501
Age	0.167
Ed	0.197
G	0.202
Emp	0.000

Note: ADHD, attention-deficit/hyperactivity disorder symptoms; ANX, anxiety symptoms; D, depressive symptoms; Ed, the level of education; Emp, employment status; ES, the exposome score; G, gender; M, manic symptoms; OCD, obsessive-compulsive disorder symptoms; PLEs, psychotic-like experiences.

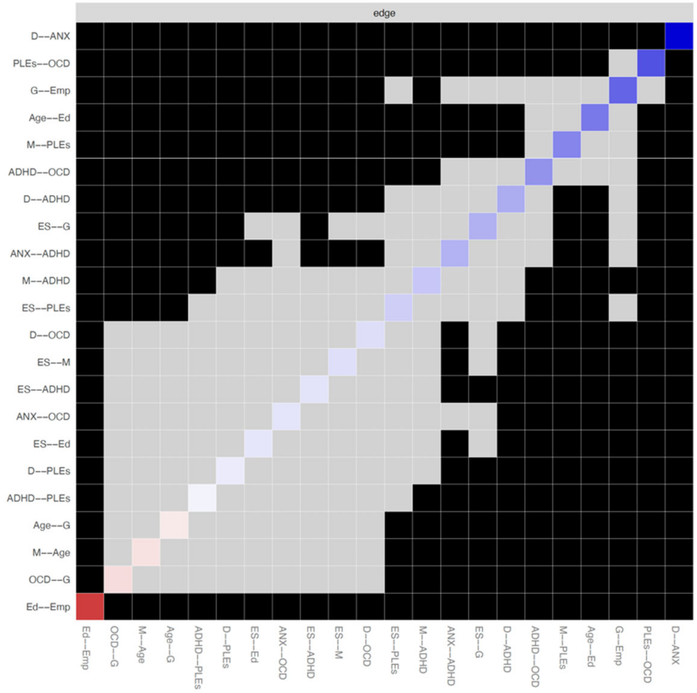

Figure S1. Bootstrapped differences between edge weights in the network analyzing symptoms associated with the exposome score. Significant differences are marked with black boxes. *Note:* ADHD, attention-deficit/hyperactivity disorder symptoms; ANX, anxiety symptoms; D, depressive symptoms; Ed, the level of education; Emp, employment status; ES, the exposome score; G, gender; M, manic symptoms; OCD, obsessive-compulsive disorder symptoms; PLEs, psychotic-like experiences.

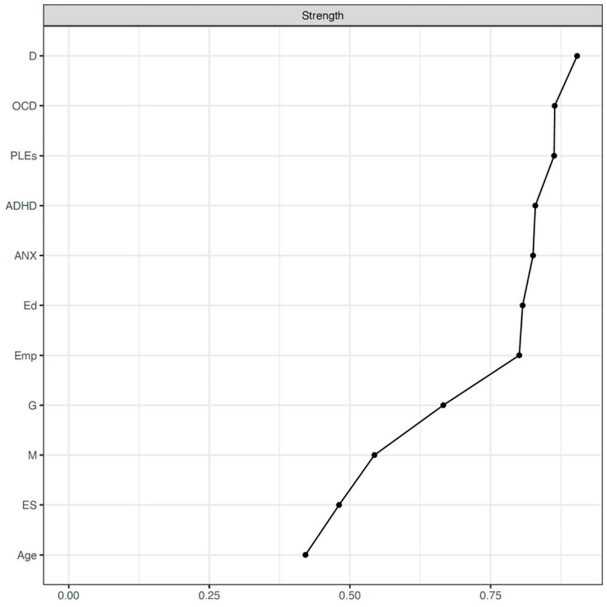

Figure S2. Strength centralities. *Note:* ADHD, attention-deficit/hyperactivity disorder symptoms; ANX, anxiety symptoms; D, depressive symptoms; Ed, the level of education; Emp, employment status; ES, the exposome score; G, gender; M, manic symptoms; OCD, obsessive-compulsive disorder symptoms; PLEs, psychotic-like experiences.

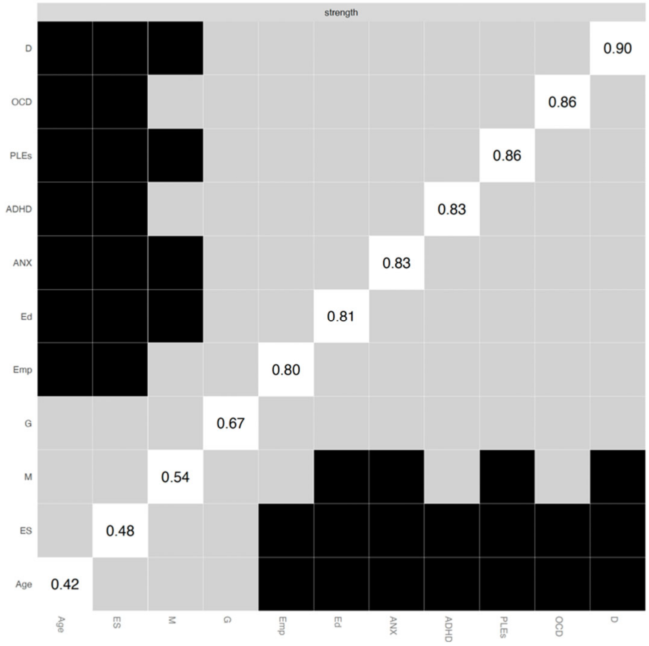

Figure S3. Bootstrapped differences in the strength centrality. Black boxed indicate significant differences. *Note:* ADHD, attention-deficit/hyperactivity disorder symptoms; ANX, anxiety symptoms; D, depressive symptoms; Ed, the level of education; Emp, employment status; ES, the exposome score; G, gender; M, manic symptoms; OCD, obsessive-compulsive disorder symptoms; PLEs, psychotic-like experiences.

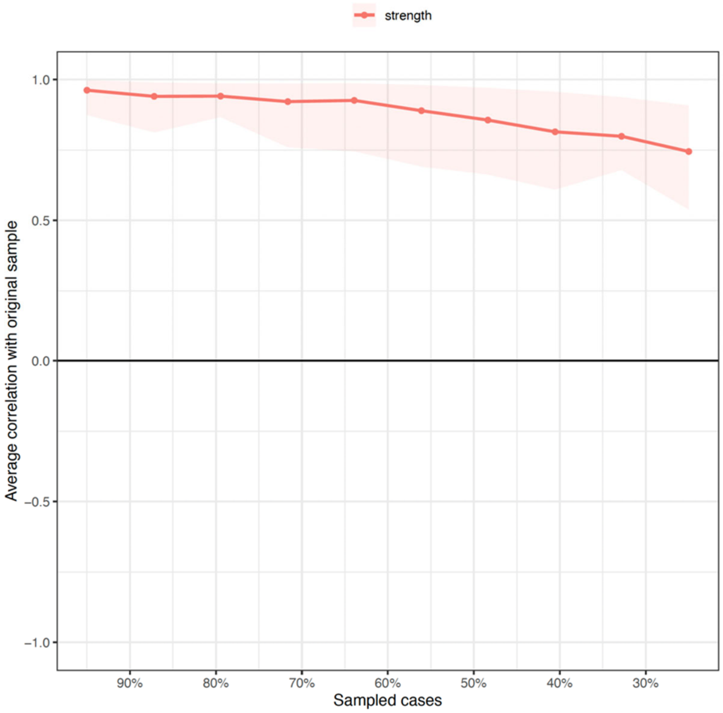

Figure S4. Stability of the strength centrality index.

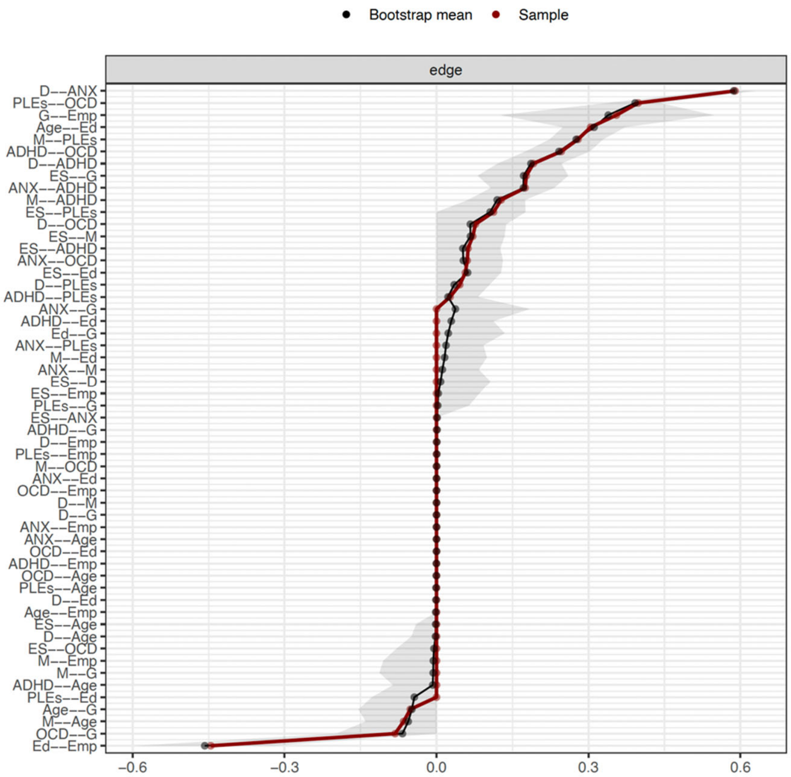

Figure S5. Bootstrapped 95% confidence intervals of edge weights. The sample values are illustrated with red lines. The black line shows bootstrapped means. The bootstrapped 95% confidence intervals are shown within the grey area. *Note:* ADHD, attention-deficit/hyperactivity disorder symptoms; ANX, anxiety symptoms; D, depressive symptoms; Ed, the level of education; Emp, employment status; ES, the exposome score; G, gender; M, manic symptoms; OCD, obsessive-compulsive disorder symptoms; PLEs, psychotic-like experiences.

References

1. Kroenke, K.; Spitzer, R.L. The PHQ-9: a new depression diagnostic and severity measure. *Psychiatric Annals* **2002**, *32*, 509-515, doi:10.3928/0048-5713-20020901-06.
2. Dumont, C.M.; Sheridan, L.M.; Besancon, E.K.; Blattner, M.; Lopes, F.; Kassem, L.; McMahon, F.J. Validity of the Mood Disorder Questionnaire (MDQ) as a screening tool for bipolar spectrum disorders in anabaptist populations. *J Psychiatr Res* **2020**, *123*, 159-163, doi:10.1016/j.jpsychires.2020.01.011.
3. Siwek, M.; Dudek, D.; Rybakowski, J.; Lojko, D.; Pawlowski, T.; Kiejna, A. [Mood Disorder Questionnaire--characteristic and indications]. *Psychiatr Pol* **2009**, *43*, 287-299.
4. Spitzer, R.L.; Kroenke, K.; Williams, J.B.; Lowe, B. A brief measure for assessing generalized anxiety disorder: the GAD-7. *Arch Intern Med* **2006**, *166*, 1092-1097, doi:10.1001/archinte.166.10.1092.
5. Ising, H.K.; Veling, W.; Loewy, R.L.; Rietveld, M.W.; Rietdijk, J.; Dragt, S.; Klaassen, R.M.; Nieman, D.H.; Wunderink, L.; Linszen, D.H.; et al. The validity of the 16-item version of the Prodromal Questionnaire (PQ-16) to screen for ultra high risk of developing psychosis in the general help-seeking population. *Schizophr Bull* **2012**, *38*, 1288-1296, doi:10.1093/schbul/sbs068.
6. Ustun, B.; Adler, L.A.; Rudin, C.; Faraone, S.V.; Spencer, T.J.; Berglund, P.; Gruber, M.J.; Kessler, R.C. The World Health Organization Adult Attention-Deficit/Hyperactivity Disorder Self-

Report Screening Scale for DSM-5. *JAMA Psychiatry* **2017**, *74*, 520-527, doi:10.1001/jamapsychiatry.2017.0298.
